# Comparison of changes in arterial blood pressure and cardiac output during cardiogenic shock development in a porcine model

**DOI:** 10.1186/s40635-025-00802-3

**Published:** 2025-09-01

**Authors:** Laura Svanekjaer, Jeppe K. P. Larsen, Peter H. Frederiksen, Louise Linde, Emilie Gregers, Nanna L. J. Udesen, Ole K. Helgestad, Ann Banke, Lisette O. Jensen, Jens F. Lassen, Amalie L. Povlsen, Henrik Schmidt, Jacob E. Møller, Hanne B. Ravn

**Affiliations:** 1 Department of Cardiothoracic Anaesthesiology, Odense University Hospital, J.B. Winsløwsvej 4, 5000 Odense, Denmark; 2 Department of Cardiology, Odense University Hospital, Odense, Denmark; 3 Department of Cardiology, Heart Center, Copenhagen University Hospital Rigshospitalet, Copenhagen, Denmark; 4 Department of Cardiothoracic Surgery, Heart Center, Copenhagen University Hospital Rigshospitalet, Copenhagen, Denmark; 5 Department of Clinical Research, University of Southern Denmark, Odense, Denmark

**Keywords:** Cardiogenic shock, Acute myocardial infarction, Arterial blood pressure, Cardiac output, Hypoperfusion

## Abstract

**Background:**

Low systolic blood pressure (SBP) is a key criterion for diagnosing cardiogenic shock (CS) caused by a reduction in stroke volume and cardiac output (CO). The temporal interaction between changes in pressure and flow has not been well described in the development of CS. In a large animal model, we assessed the temporal relationships of SBP, CO, and blood flow in the carotid artery during induction of CS.

**Methods:**

Fifteen adult Danish landrace pigs (median weight 71 kg) underwent CS induction by stepwise injection of polyvinyl alcohol microspheres into the left main coronary artery every 3 min to induce microvascular obstruction. After each injection, CO, SBP, and mixed venous saturation (SvO_2_) were recorded simultaneously from a ventricle sheath in the carotid artery and a pulmonary artery catheter in the right internal jugular vein. A Doppler flow probe measured blood flow in the left carotid artery. CS was defined as a ≥ 50% reduction in CO or SvO_2_ from baseline, or absolute SvO_2_ < 30%.

**Results:**

CS occurred after a mean of 8 (range 5 to 19) boluses of microspheres. SBP declined from 99 (± 15) mmHg to 74 (± 6) mmHg, equal to 74 (± 13)% of the baseline value. CO was reduced to 5.8 (± 0.7) L/min to 2.2 (± 1.3) L/min, equal to 38 (± 23)% and SvO_2_ from 63 (± 7)% to 37 (± 7)%, equal to 60 (± 13)% of baseline values. The decrease in CO was due to a reduction to 43 (± 26)% in stroke volume, as heart rate remained unchanged. The carotid artery blood flow was reduced from 285 (± 50) mL/min to 155 (± 56) mL/min, equal to 54% of baseline values. The decline in SvO_2_ and CO preceded a reduction in SBP, and after 25% of emboli were given, CO decreased by 24% while SBP was unchanged.

**Conclusion:**

In a porcine model of ischemic myocardial injury, the decrease in blood flow and stroke volume preceded a decline in SBP, suggesting pressure preservation occurs in the presence of hypoperfusion.

**Supplementary Information:**

The online version contains supplementary material available at 10.1186/s40635-025-00802-3.

## Introduction

Acute myocardial infarction (AMI) is characterized by loss of myocardial contractility. In more severe cases, it leads to a critical reduction in cardiac output (CO) and development of cardiogenic shock (CS). CS develops in 5–10% of AMI patients and is associated with a mortality rate exceeding 40% [1, 2].

CS is defined as hypotension (or need for vasopressor) in combination with clinical signs of impaired tissue perfusion with cold and clammy extremities, altered mental status, oliguria, and/or an increased lactate level [3–5]. However, symptoms and shock severity vary considerably, which has led to the introduction of a classification scheme by the Society for Cardiovascular Angiography and Intervention (SCAI). The SCAI shock classification is a five-stage system (A–E) that stratifies CS severity, ranging from Stage A (At Risk) with no overt shock to Stage E (Extremis), guiding risk assessment, management, and prognosis [6]. The classification system recognizes the dynamic nature of the condition, with classes A and B identifying patients at risk who are beginning to lose compensatory mechanisms. Although CS per definition is a consequence of low CO, the most readily quantifiable measure is the systolic blood pressure (SBP), which also is a key component in the SCAI classification [7, 8]. When acute ischemia reduces left ventricular (LV) contractility, it will cause a decline in stroke volume and ultimately a reduction in CO. However, some patients may remain normotensive despite suffering hypoperfusion [9], and little is known about the temporal interdependence of pressure and flow during CS progression from SCAI A to C.

The aim of the present study was to describe simultaneous changes in blood pressure and CO during progressive myocardial injury after repeated intracoronary microsphere injections in a porcine model of CS.

## Methods

This translational animal study included 15 Danish landrace, female pigs at the age of 18 weeks with a median weight of 71 kg; all animals originated from a single certified farmer on Fyn, Region of Southern Denmark. The animal experiments were conducted in adherence with the rules and regulations of the Danish Animal Experiment Inspectorate and Animal Research: Reporting of In Vivo Experiments (ARRIVE) guidelines and approved under Study ID: 2006–15-00951. All animals were housed and cared for according to Danish law regarding animal studies and euthanized at the end of the study [10]. To acclimate, the animals arrived one week prior to the study.

This is an exploratory study based on a previous intervention study [11], and the present study comprised all animals with available data prior to mechanical circulatory support randomization. Animals were anesthetized with sevoflurane and fentanyl infusion (25–50 μg/kg/h) and ventilated with an oxygen fraction of 30–50%, a tidal volume of 6–8 mL/kg, a rate of 10–14/min, and a positive end-expiratory pressure of 5 cm H_2_O with adjustment prior to baseline measurements as previously described [12]. During CS induction, no ventilator adjustments were performed. An initial intravenous amiodarone bolus of 300 mg and a maintenance infusion of 50 mg/h were administered to prevent malignant arrhythmias.

The left main coronary artery was catheterized using a JL 3.5 catheter (Launcher, Medtronic Inc., Minneapolis, MN, USA) through a 7 Fr. sheath in the right femoral artery to inject microspheres. Each injection consisted of 1 ml of a mixture of 125 µg of polyvinyl alcohol flakes (Contour™; Boston Scientific, Marlborough, USA) dissolved in 10 ml isotonic saline and 10 ml of radiopaque contrast. Embolization was repeated every 3 min until CS was established, and simultaneous measurements of hemodynamic variables and conductance catheter data were recorded 90 s after each injection. CS was defined as a ≥ 50% reduction in CO or mixed venous saturation (SvO_2_) compared to baseline or an absolute SvO2 < 30%. Blood gas analysis with lactate was performed at baseline and at manifest CS. Norepinephrine infusion could be initiated as part of the original protocol if mean arterial pressure (MAP) decreased below 50 mmHg before CS was established. For the present study, animals treated with norepinephrine (N = 4) were censored at the time of initiation of norepinephrine [13].

### Hemodynamic assessment

The left carotid artery was exposed by surgical cut-down, and a 4-mm Doppler flow probe (MEDISTIM SonoQ TTFM Probe, Emtec GmbH, Finning Germany) measured continuous carotid blood flow. A pulmonary artery catheter was inserted in the right internal jugular vein for continuous measurements of SvO_2_ and pulmonary artery pressures. In the right carotid artery, an 8 Fr. sheath was inserted for continuous measurements of systemic arterial blood pressure and for advancing a conductance catheter retrogradely into the LV (Ventri-cath 510 PV Loop Catheter, Millar Inc. TX, USA). The conductance catheter was connected to a MPVS Ultra Pressure–Volume loop (PVL) system (Millar Inc, TC, USA). Data sampling was performed by connecting a Powerlab 16/35, and analysis was performed in LabChart pro (ADInstruments, Dunedin). With electrocardiogram electrodes connected to Powerlab 16/35, heart rate was measured. To determine parallel wall conductance, an infusion of hypertonic saline was used. With the pulmonary artery catheter thermodilution derived CO, the alpha calibration was calculated. An occlusion of the inferior vena cava was performed to reduce preload to determine the estimated ventricular volume at zero pressure (V_0_). V_0_ was calculated with linear fit and kept constant throughout the measurements in the experiment. From the pressure–volume loop, the following parameters were recorded: LV end-diastolic pressure (LVEDP), LV end-systolic pressure (LVESP), LV ejection fraction (LVEF), and stroke work. CO was calculated as stroke volume multiplied by heart rate.

### Statistics

Data are presented as mean with standard deviation for absolute values of all hemodynamic and conductance variables after evaluation of normal distribution by visual assessment, QQ-plots, and histograms. Since the number of microsphere injections varied between animals, the percentage of the total number of emboli was calculated and presented as baseline equal to 0% and CS equal to 100%. In Table 1, a mean was calculated for 1–25%, 26–50%, 51–75%, and 76–99% of the number of emboli.

**Table 1:** Hemodynamic changes during injections of microspheres

	Baseline (n = 11)	1–25% (n = 10)	26–50% (n = 8)	51–75% (n = 8)	76–99% (n = 8)	Cardiogenic shock (n = 8)	p-value
Hemodynamics							
Systolic blood pressure (mmHg)	99 (15)	95 (12)	89 (7)*	84 (7)*	74 (8)*	74 (6)*	< 0.001
Diastolic blood pressure (mmHg)	58 (11)	55 (11)	51 (10)*	51 (9)*	46 (9)*	45 (7)*	< 0.001
MAP (mmHg)	74 (12)	70 (12)	64 (9)*	62 (9)*	55 (9)*	55 (7)*	< 0.001
CVP (mmHg)	12 (4)	13 (4)	14 (3)	15 (3)*	15 (3)*	14 (3)*	0.001
Heart rate (beats per min.)	78 (11)	74 (9)*	73 (9)*	71 (9)*	71 (9)*	71 (9)*	< 0.001
SvO_2_ (% saturation)	63 (7)	59 (9)	53 (7)*	46 (8)*	39 (7)*	37 (7)*	< 0.001
Carotid blood flow (mL/min)	285 (50)	236 (70)*	215 (57)*	185 (47)*	158 (49)*	155 (56)*	< 0.001
Carotid blood flow (% change)	100	83*	76*	65*	56*	54*	< 0.001
Conductance catheter							
CO (L/min.)	5.8 (0.7)	4.4 (0.8)*	3.6 (0.9)*	3 (1)*	2.7 (1) *	2.2 (1.3)*	< 0.001
Stroke volume (mL)	75 (10)	61 (10)*	50 (11)*	43 (16)*	40 (16)*	32 (19)*	< 0.001
LVEDP (mmHg)	20 (2)	22 (2)*	23 (2)*	23 (2)*	24 (2)*	24 (2)*	< 0.001
LVESP (mmHg)	92 (11)	79 (10)*	78 (9)*	71 (8)*	67 (7)*	61 (10)*	< 0.001
LVEF (%)	43 (7)	29 (7)*	25 (8)*	21 (10)*	19 (10)*	16 (10)*	< 0.001
Stroke work (mmHg*mL)	5726 (1212)	3569 (1070)*	2805 (790)*	2089 (986)*	1757 (850)*	1287 (1062)*	< 0.001

Data are presented as mean (± SD). P-value of linear mixed-effects model. * P < 0.01 in comparison with baseline.

*MAP* mean arterial pressure, *CVP* central venous pressure, *SvO*_*2*_ mixed venous saturation, *CO* cardiac output, *LVEDP* left ventricular end-diastolic pressure, *LVESP* left ventricular end-systolic pressure, *LVEF* left ventricle ejection fraction

The analysis of temporal variations and significance levels in hemodynamic parameters was done using a linear mixed-effects model, with percentage of emboli incorporated as a categorical variable at baseline to 1–25%, 26–50%, 51–75%, 76–99% shock, and the point of manifest CS, as this analysis permitted incorporation of fixed and random effects to address within-subject variability and missing or unbalanced data. If the overall mixed linear analysis was significant, additional comparisons were performed with each quartile of emboli given in comparison to baseline. A p-value less than 0.05 was considered statistically significant. All statistical analyses were conducted using R Statistical Software version 4.4.1 (R Core Team 2024).

## Results

Among 15 animals, one was excluded due to cardiac arrest (asystole), one due to technically inadequate conductance data, and two due to norepinephrine infusion from the beginning of microembolization, leaving 11 animals at baseline available for analysis. After 25% of intracoronary microsphere injections, an additional two animals were excluded due to the initiation of norepinephrine infusion to maintain MAP > 50 mmHg (Table 1).

Before CS was established, an average of 8 (range 5 to 19) boluses of intracoronary microspheres were given.

**Fig. 1:**
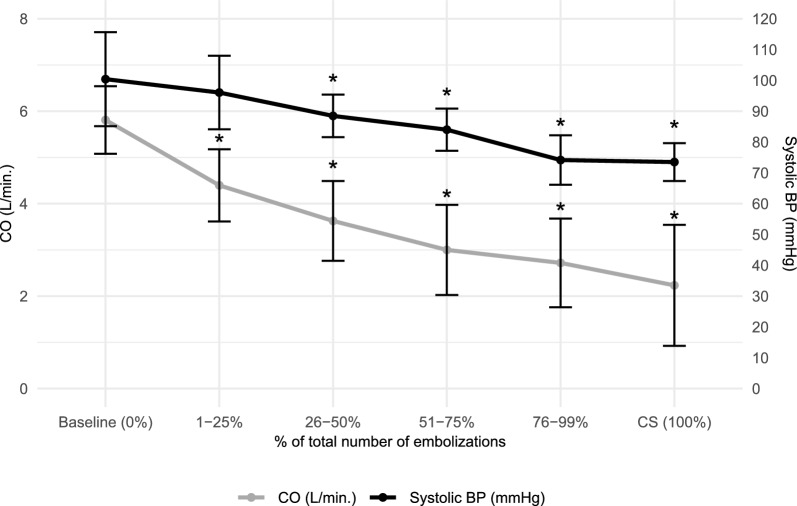
Plot of changes in systolic blood pressure and cardiac output during embolization. Plot of mean ± SD of *CO* cardiac output and *SBP* systolic blood pressure from baseline to manifest *CS* cardiogenic shock. Significance compared to baseline values: * P < 0.01

**Fig. 2:**
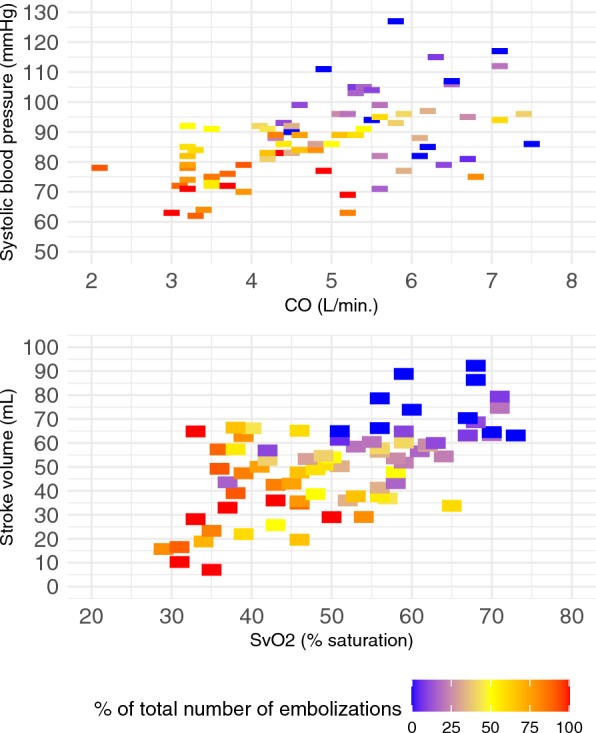
Heatmaps of hemodynamic changes during embolization. Heatmap of systolic blood pressure, cardiac output, stroke volume, and mixed venous saturation. Color graded with the percentage of the total number of emboli. CO: cardiac output, SvO_2_: mixed venous saturation.

The slope of decline in SBP and CO had markedly different trajectories (Fig. 1). Based on slope estimates from the linear mixed-effects model, CO decreased by 0.66 L/min, and SBP by 6.39 mmHg per percent increase in emboli. The percentage of emboli given to each individual animal is shown by color gradients in heatmaps (Fig. 2) to visualize the correlation between SBP and CO as well as stroke volume and SvO_2_.

When CS was established, SBP was reduced from 99 (± 15) mmHg to 74 (± 6) mmHg, equal to 74 (± 13)% of baseline level (Table 1; *p* < 0.001). MAP was equally reduced to 75 (± 15)% of baseline. CO decreased in the same time period from 5.8 (± 0.7) L/min to 2.2 (± 1.3) L/min, equal to 38 (± 23)% of baseline level (Table 1; *p* < 0.001). After one-quarter of intracoronary microspheres boluses were given, CO had decreased to 4.4 (± 0.8) L/min, equal to 76% of the baseline level, *p* < 0.001, whereas SBP and MAP remained unchanged at 95 (± 15) mmHg from 99 (± 12) mmHg and 70 (± 12) mmHg from 74 (± 12) mmHg, respectively (Fig. 2, Table 1). The decrease in CO was almost exclusively caused by a reduction in stroke volume from 75 (± 10) mL to 32 (± 19) mL, equal to 43 (± 26)%; whereas, heart rate remained unchanged from 78 (± 11) beats per min to 71 (± 9) beats per min (Table 1). Reduction in CO was associated with a decrease in absolute SvO_2_ values from 63 (± 7)% to 37 (± 7)%, equal to 60 (± 13)% of the baseline value when CS was established. In agreement, LVEF in absolute percentage was reduced from 43 (± 6)% to 16 (± 10)%. Arterial lactate increased from 1.1 (± 0.4) mmol/L to 3.0 (± 1.8) mmol/L. Carotid artery blood flow decreased from 285 (± 50) mL/min to 155 (± 56) mL/min, equal to 54% of baseline and was thus better preserved than CO at the time of CS.

**Fig. 3:**
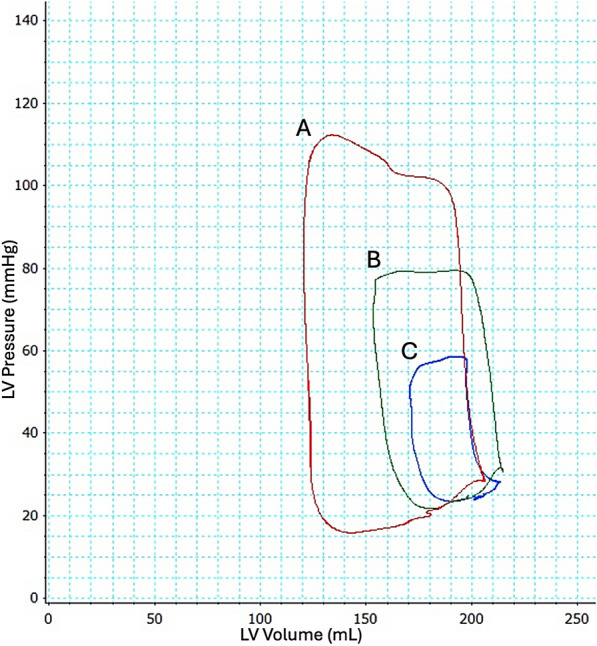
Pressure–volume changes during embolization. Example of pressure–volume loop changes in the left ventricle (LV) at red (**A**); baseline, green (**B**); 50% CS and blue (**C**); manifest cardiogenic shock. Cardiac output is 5.825 L/min, 4.297 L/min, and 2.147 L/min, respectively

Representative pressure–volume loops during CS induction are displayed in Fig. 3, demonstrating a significant decline in stroke volume and LV pressure generation.

## Discussion

In this translational animal study with myocardial injury induced by intracoronary microspheres injection into the left main coronary artery in adult pigs, CO decreased faster and the magnitude exceeded the decrease of SBP when CS was fully established. When one-quarter of emboli had been given, CO decreased by 25% while SBP remained unchanged. Carotid artery blood flow was better preserved than CO during development and at the time CS was established.

### The definition of CS

The classic hallmarks of CS include a low SBP caused by low CO despite adequate filling and systemic hypoperfusion. In the clinical setting, identification of CS can be difficult, since measurements of CO and filling pressures require invasive measurements or advanced echocardiography [14]. Therefore, the clinical diagnosis will often rely on low systemic blood pressure, signs of congestion, and organ hypoperfusion, i.e., cold extremities, altered mental status, oliguria, as well as anaerobic metabolism measured as an increased lactate level in blood [3, 4, 14].

In the present study, CO decreased with 24% after the first quarter of intracoronary microsphere injections, but SBP remained unchanged. In the clinic, a patient with both hypotension and hypoperfusion reflects the classic SCAI stage C CS, and these patients have a higher mortality risk than patients presenting with hypoperfusion but no hypotension. However, patients with hypoperfusion but no hypotension have a higher mortality risk compared to patients presenting with isolated hypotension but no sign of hypoperfusion (SCAI stage B) [15]. Since CO is not easily measurable, a significant decrease can be present despite preserved SBP. This may contribute to a delayed identification of unstable hemodynamics after AMI leading to a worse outcome [16] due to an extended period with hypoperfusion prior to a targeted intervention to alleviate the low CO [6]. This was demonstrated by Pilarczyk et al. in almost 25% of the patients presenting without hypotension in the early stages of CS [17].

This inherently leads to the question of optimizing the threshold of SBP to identify patients early in CS, but without including too many patients. The most frequently used threshold of SBP in the diagnosis of CS is < 90 mmHg, but thresholds < 100 or < 80 mmHg have also been applied [16, 18, 19]. The fact that blood pressure is a dynamic parameter influenced by several components, including comorbidities, pain, anxiety, drugs, etc., it is important to remind the clinician that CO may be more jeopardized than the blood pressure level indicates during CS development. In short, an acceptable SBP is not always equivalent to an acceptable CO [20]. Therefore, in patients with signs of hypoperfusion, additional diagnostic tools should be applied, including ultrasound to assess LV outflow tract velocity time integral and arterial pressure analysis in the setting of AMI [14, 20].

### The cerebral blood flow

Peripheral vasoconstriction occurs when CO decreases to maintain perfusion pressure and centralization of flow to vital organs as previously reported [3, 5, 14, 21, 22], and confirmed in this study, as carotid artery blood flow is better preserved.

In CS, the decrease in CO is accompanied by increased systemic vascular resistance centralizing blood flow to vital organs [23]. This can cause regional hypoperfusion while preserving flow to other organs. The cerebral blood flow can be affected by both changes in blood flow and blood pressure [23, 24], but due to cerebral autoregulation, it is more resilient to changes in pressure. Accordingly, blood flow in the carotid artery was better preserved despite a significant reduction in CO. This is in agreement with observations from Yahagi et al., who used an in vitro circuit exclusive of the right ventricle. Yet, hemodynamic differences have to be considered due to the in vitro design by Yahagi et al. compared to this in vivo animal model [21].

### Limitations

Animal studies can contribute to a better pathophysiological understanding of the development of CS during standardized conditions to give a better signal-to-noise ratio in hemodynamic observations [21]. Pig cardiovascular anatomy and physiology share many similarities with humans, including end-artery coronary anatomy [20, 25]. However, the pigs used in the present study were young and healthy, unlike CS patients who are often older and suffering from comorbidities, and the duration of the low perfusion period may have been shorter than in patients. Therefore, some results may not translate into clinical practice. As the animals served as their own controls, a control group was not included. To prevent ventricular fibrillation, all pigs received an intravenous infusion of amiodarone prior to microembolization. Amiodarone may have prevented compensatory sinus tachycardia, which consequently also can have led to a faster induction of CS. To prevent myocardial hypoperfusion due to critical hypotension, some animals received norepinephrine during CS induction to keep MAP > 50 mmHg. Since norepinephrine increases systemic vascular resistance, it abolishes the natural association between flow and pressure [26]. Therefore, norepinephrine-treated animals were excluded from analysis from the time of administration (data including norepinephrine-treated animals can be found in Supplementary). Carotid blood flow was assessed using Doppler flow, and despite angle adjustment to give a more precise measurement [23], variation in size and flow in the carotid artery between animals and movement of the probe cannot be ruled out. As the distribution of blood flow in the vertebral and carotid arteries is unknown, carotid artery blood flow cannot be directly translated into cerebral blood flow.

## Conclusion

In a porcine model of CS, CO and stroke volume decreased faster and to a greater extent compared to systemic arterial blood pressure during CS induction. Blood flow in the carotid artery was better preserved than CO, possibly due to cerebral autoregulation.

In a clinical setting, these observations emphasize the importance of considering CS in patients showing signs of hypoperfusion, even if their SBP appears normal.

## Take home message

This study found a decrease in blood flow and stroke volume preceding the decrease of arterial blood pressure during ischemic myocardial injury in a porcine model. Cardiac output may be more jeopardized compared to systolic blood pressure before cardiogenic shock is evident.

## Supplementary Information

Supplementary file 1.

## Data Availability

The data used and analyzed during the current study are available from the corresponding author on reasonable request.

## Abbreviations

AMI: Acute myocardial infarction
CO: Cardiac output
CS: Cardiogenic shock
CVP: Central venous pressure
LV: Left ventricular
LVEDP: Left ventricular end-diastolic pressure
LVEF: Left ventricular ejection fraction
LVESP: Left ventricular end-systolic pressure
MAP: Mean arterial blood pressure
SBP: Systolic blood pressure
SCAI: Society for cardiovascular angiography and intervention
SvO_2_: Mixed venous saturation
