# Supplementary material for: Comparison of changes in arterial blood pressure and cardiac output during cardiogenic shock development in a porcine model
Source: Intensive Care Med Exp. 2025 Sep 1;13:91. doi: 10.1186/s40635-025-00802-3 (PMC12401854; doi:10.1186/s40635-025-00802-3)
Supplement: Supplementary file 1 [file 40635_2025_802_MOESM1_ESM.docx]

**Table 1 Supplementary – Hemodynamic changes during injections of microspheres including animals receiving norepinephrine.**

|  | **Baseline (n=13)** | **1-25% (n=12)** | **26-50% (n=10)** | **51-75% (n=10)** | **76-99% (n=10)** | **Cardiogenic shock (n=10)** | **p-value** |
| --- | --- | --- | --- | --- | --- | --- | --- |
| **Hemodynamics** | | | | | | |  |
| **Systolic blood pressure (mmHg)** | 96 (16) | 93 (12) | 86 (10)* | 85 (6)* | 75 (7)* | 72 (6)* | <0.001 |
| **Diastolic blood pressure (mmHg)** | 56 (12) | 54 (11) | 48 (10)* | 51 (8)* | 45 (8)* | 43 (7)* | <0.001 |
| **MAP (mmHg)** | 71 (12) | 69 (12) | 6 (10)* | 63 (8)* | 55 (8)* | 53 (7)* | <0.001 |
| **CVP (mmHg)** | 12 (4) | 13 (4) | 14 (3) | 14 (3)* | 14 (2)* | 14 (2)* | <0.001 |
| **Heart rate (beats per min.)** | 76 (11) | 73 (8) | 72 (8) | 72 (8) | 72 (9) | 71 (8) | 0.006 |
| **SvO_2_ (% saturation)** | 62 (7) | 59 (10) | 49 (10)* | 46 (8)* | 38 (7)* | 36 (8)* | <0.001 |
| **Carotid blood flow (mL/min)** | 26 (67) | 234 (63) | 212 (54)* | 197 (48)* | 166 (50)* | 154 (51)* | <0.001 |
| **Carotid blood flow (% change)** | 100 | 88 | 80* | 74* | 63* | 58* | <0.001 |
|  | **Baseline (n=13)** | **1-25% (n=12)** | **26-50% (n=10)** | **51-75% (n=10)** | **76-99% (n=10)** | **Cardiogenic shock (n=10)** | **p-value** |
| **Conductance catheter** | | | | | | |  |
| **CO (L/min.)** | 5.6 (0.8) | 4.4 (0.7)* | 3.8 (0.9)* | 3.3 (1.2)* | 3 (1)* | 2.2 (1.2)* | <0.001 |
| **Stroke volume (mL)** | 74 (10) | 60 (9)* | 53 (12)* | 47 (17)* | 42 (16)* | 32 (17)* | <0.001 |
| **LVEDP (mmHg)** | 20 (2) | 21 (3)* | 22 (2)* | 23 (2)* | 23 (2)* | 24 (2)* | <0.001 |
| **LVESP (mmHg)** | 89 (14) | 76 (12)* | 76 (10)* | 71 (7)* | 65 (7)* | 58 (12)* | <0.001 |
| **LV EF (%)** | 43 (6) | 30 (8)* | 27 (9)* | 22 (10)* | 21 (10)* | 16 (9)* | <0.001 |
| **Stroke work (mmHg*mL)** | 5438 (1316) | 3423 (1067)* | 2890 (844)* | 2311 (1049)* | 1842 (796)* | 1182 (962)* | <0.001 |

Data are presented as mean (±SD). P-value of linear mixed effects model. * P < 0.01 in comparison with baseline.

MAP: mean arterial pressure, CVP: central venous pressure, SvO_2_: mixed venous saturation, CO: cardiac output, LVEDP: Left ventricular end diastolic pressure, LVESP: Left ventricular end systolic pressure, LVEF: Left ventricle ejection fraction
